# Nature’s Synergy: Cellular and Molecular Evaluation of Snail Slime and Its Principal Component, Glycolic Acid, on Keratinocytes, with Preliminary Evidence from Endothelial Cells

**DOI:** 10.3390/biom15091302

**Published:** 2025-09-10

**Authors:** Muhammad Rashad, Alessia Ricci, Serena Pilato, Amelia Cataldi, Marwa Balaha, Susi Zara

**Affiliations:** 1 Department of Pharmacy, “G. d’Annunzio” University of Chieti-Pescara, 66100 Chieti, Italy; muhammad.rashad@unich.it (M.R.); alessia.ricci@unich.it (A.R.); serena.pilato@unich.it (S.P.); amelia.cataldi@unich.it (A.C.); marwa.balaha@unich.it (M.B.); 2 UdA-Tech Lab Research Center, “G. d’Annunzio” University of Chieti-Pescara, 66100 Chieti, Italy; 3 Department of Pharmaceutical Chemistry, Faculty of Pharmacy, Kafrelsheikh University, Kafrelsheikh P.O. Box 33516, Egypt

**Keywords:** Snail slime, wound healing, matrix metalloproteinases, inflammation, glycolic acid, ROS

## Abstract

Snail slime (SS) is a natural secretion rich in bioactive components such as glycoproteins, hyaluronic acid, glycolic acid (GA), and antimicrobial peptides. GA, a key component of SS, is known for its exfoliative properties. This study investigates SS’s effects on keratinocytes (HaCaT) and endothelial cells (ECs), comparing its properties to those of GA. HaCaT cell viability and cytotoxicity, ROS release, and inflammation-related signaling (PI3K/Akt/NF-κB and COX-2 gene expression) were assessed. Extracellular matrix (ECM) remodeling was evaluated by gene expression of MMPs. In ECs, a preliminary evaluation of SS’s effect was conducted in terms of cell viability and migration. Results demonstrated that SS is well tolerated by keratinocytes whereas GA exhibits cytotoxicity, suggesting that SS’s natural composition mitigates GA’s adverse effects. SS induced a controlled, brief inflammatory response, via the PI3K/Akt/NF-κB pathway, unlike GA, responsible for stronger and sustained pro-inflammatory events. Additionally, SS, through the upregulation of MMPs, contributes to ECM remodeling. In ECs, SS preserves viability and also enhances migration, thus supporting wound healing. These findings highlight SS’s ability to balance pro-inflammatory events, making it a promising candidate for advanced dermatological applications, underscoring SS’s potential in modulating key cellular signaling pathways, and supporting its future therapeutic prospects in wound healing.

## 1. Introduction

Snail slime (SS), also referred to as snail mucus or snail mucin, is a complex viscoelastic fluid secreted by gastropod mollusks (e.g., *Helix aspersa*) which has garnered significant scientific interest due to its unique composition and potential applications [1]. Although it is primarily composed of water, it contains a diverse array of biologically active compounds [2,3] that contribute to the slime’s multifunctional role in gastropod biology, facilitating locomotion, adhesion, and protection against desiccation and predators, and it even plays a role in reproduction [4,5].

SS has a complex composition, including water, the main component of SS, comprising up to 90–99.7% of its total weight [6] while in dry form it includes proteins [7], glycoproteins (mucins, lectins), polysaccharides (glycosaminoglycans), lipids, metal ions, aromatic amino acids [8], allantoin, collagen, elastin, glycolic acid (GA), achacin, antioxidants, hyaluronic acid, and peptides. These components contribute to its well-documented anti-inflammatory, antimicrobial, anti-aging, and antioxidant activities [9].

Each component contributes to the slime’s regenerative and protective properties. For instance, hyaluronic acid is a powerful humectant, drawing moisture to the skin and helping maintain hydration [10]. Allantoin is known for its anti-inflammatory, soothing, and healing properties [11], while proteins and peptides in the mucin may support collagen production and skin elasticity [12]. GA, the smallest molecule in the family of α-hydroxy acids, has a keratinolytic effect acting as a gentle exfoliant, promoting cell turnover and improving skin texture [13]. GA is a nontoxic water-soluble white crystalline powder with a pH of 1.7 at 5% concentration. When used below 10%, GA is considered safe and effective in promoting skin regeneration by upregulating the synthesis of glycosaminoglycans and by stimulating collagen production [14]. However, its effects vary based on contact time, concentration, and availability of free acid. Prolonged exposure exceeding 10 min has been associated with skin irritation and inflammation [15].

Thanks to this unique combination of components, SS offers a powerful synergy of moisturizing, exfoliating, and healing effects; its components, in fact, work synergistically to promote skin regeneration, boost collagen production, and provide powerful antioxidant effects [16].

The unique properties of SS have sparked interest in various fields, particularly biomedicine, pharmaceutical industries, and cosmetics, which are currently exploring and exploiting snail mucin for its moisturizing, anti-aging, and skin-repair properties [17,18] In particular, in biomedicine, components like allantoin and hyaluronic acid show promise in wound healing and tissue regeneration [19,20], while antimicrobial peptides present in SS may serve as candidates for novel antimicrobial agents [21].

Human skin is composed of epidermis, dermis, and subcutaneous tissue. The epidermis is the outermost layer, primarily consisting of keratinocytes, while the lower dermis, with loose connective tissue with fibroblasts, contains blood vessels in which endothelial cells (ECs) are also represented. The epidermis is involved in skin regeneration by replacing the outer dead keratinocytes with new cells and the dermis supports wound healing by continuously remodeling the ECM and promoting new blood vessel formation [22]. Moreover, keratinocytes are also involved in the initiation of skin inflammation by specialized keratinocyte-intrinsic mechanisms [23].

Inflammation, in fact, can be considered a defensive mechanism in response to any internal or external damage to the skin or body organ. Inflammation can be beneficial or harmful depending on the cause and duration. Chronic inflammation is harmful while acute or brief inflammation is a part of the immune response and can be helpful in many ways. For example, for injuries or wounds, the release of cytokines and recruitment of macrophages to clean the debris, followed by cell migration and re-epithelialization to the site of injury [24], represents a key passage for the whole wound healing and recovery process.

In our previous study, we tested SS on fibroblasts and the results evidenced SS’s capability to promote fibroblast viability and to trigger recovery mechanisms by activating the Erk protein. Moreover, an appreciable anti-inflammatory effect due to the significant reduction in cyclooxygenase-2 expression (COX-2) and the positive modulation of new blood vessel formation demonstrated by increased Angiopoietin 1 gene expression and a higher matrix deposition (evidenced by the augmented amount of released collagen I) could be identified [20]. Thus, based on this knowledge, this study aimed at deepening the effect of SS on the outermost skin layer, i.e., keratinocytes, represented by epidermidis. Considering that GA is one of the main components of SS, we compared, for the first time, the effect of SS to that obtained by administering GA alone, elucidating the main differences in triggering the pro-inflammatory event that could be essential to promote skin exfoliation and re-epithelization. Starting from the assumption that an appreciable reparative/regenerative effect within the epidermidis takes place only if a functioning blood vessel system in the dermis exists, a further and initial evaluation of SS biocompatibility on ECs was also performed, in order to estimate SS’s possible capability to support ECs and their migration, thus indirectly sustaining angiogenic events.

## 2. Materials and Methods

### 2.1. SS Extraction

*Helix aspersa’s* slime was extracted using the cruelty-free Cherasco Muller method at Lumacheria Italiana srl (Cherasco, Italy), as already reported elsewhere [2,6,20]. The Cherasco Muller method focuses on animal welfare, preserving animal life and minimizing the risk of endangered species. Additionally, this method allows multiple extractions (up to 3) using the same animals with a gap of minimum two weeks to achieve better SS composition. The method is approved by the relevant authorities in charge, which not only monitor animals’ health but also provide them an optimum environment for reproduction. Briefly, adult and healthy snails were recruited, washed in running water at room temperature, and transferred to a Muller extractor. A Muller extractor is an automatic machine with an installed software set for antimicrobial washing and awakening of snails (30 min) using ozonated water followed by three cycles (10 min each) of 10% citric acid-stimulated slime extraction. The collected SS was then filtered through 0.2-micron filter and 0.1% of both sodium benzoate and potassium sorbate preservatives were mixed to filtered SS to enhance shelf life and sustain quality.

### 2.2. Cell Culture

A human keratinocyte (HaCaT) cell line (catalog # T0020001), purchased from AddexBio (AddexBio, San Diego, CA, USA), and somatic hybrid ECs from the human umbilical vein (EA.hy926), purchased from the American Type Culture Collection (ATCC), CRL-2922 (p10), were both cultured in high-glucose Dulbecco’s Modified Eagle Medium (DMEM), supplemented with 1% penicillin/streptomycin and 10% Fetal Bovine Serum (FBS) at 37 °C with 5% CO_2_.

### 2.3. Cell Treatments

HaCaT cells were seeded at 25,000 cells/cm^2^ in tissue-culture-treated multiwell plates. EA.hy926 cells were seeded at 8000/well in a 96-well plate and 80,000/well in a 12-well plate to perform wound healing. Both cell types were cultured in DMEM.

After an overnight incubation, both HaCaT and EA.hy296 cells were treated with SS dilutions of 1:40 (0.508 mg/mL), 1:60 (0.31 mg/mL), and 1:80 (0.247 mg/mL) and GA dilutions of 4.1 mM (0.31 mg/mL), 2.73 mM (0.21 mg/mL), and 2.05 mM (0.15 mg/mL) in DMEM while the control was represented by cells only receiving potassium sorbate and sodium benzoate preservatives at 0.1% and sodium citrate at 10% in DMEM, to normalize the effect of stabilizers. These concentrations were chosen according to the results obtained in the previous paper [20].

### 2.4. MTT Assay

Cell viability in HaCaT cells was measured after 24 and 48 h of treatment in the presence of SS (1:40, 1:60, 1:80) and GA (4.1, 2.73, and 2.05 mM), while in EA.hy926 cells, it was measured after 24, 48, and 72 h of treatment with SS (1:40, 1:60, 1:80) and GA (4.1 mM), by an MTT (3-(4,5-dimethylthiazol-2-yl)-2,5-diphenyltetrazolium bromide) assay (Merck Life Science, Milan, Italy). After the prescribed time, the culture medium was removed and refed with 10% MTT (0.5 mg/mL) in DMEM (100 μL/well) and incubated for 4 h at 37 °C with 5% CO_2_ in the dark. After that, the same amount of DMSO (100 μL/well) was added to dissolve the formazan crystals, and again, the mixture was incubated for 20 min at 37 °C. The colored solution obtained by the dissolution of formazan crystals was an indication of the presence of viable cells. The plate was read at 540 nm using a GO microplate spectrophotometer (Thermo Fisher Scientific, Waltham, MA, USA) and the percentage of metabolically active cells was obtained by comparing the sample with the control, whose viability was established at 100%.

### 2.5. Lactate Dehydrogenase (LDH) Cytotoxicity Assay

Cytotoxicity was measured after 14 and 24 h of SS (1:40, 1:60, 1:80) and GA (4.1 mM, amount present in most concentrated dilution of SS (1:40)) treatment of HaCaT cells by using the CytoTox 96 non-radioactive cytotoxicity assay (Promega, Madison, WI, USA). It worked by measuring the LDH leakage from cells after treatment with SS and GA. The experiment was performed following the manufacturer’s instructions and optical density (OD) was measured at 490 nm with a background absorbance of 690 nm. Measured LDH leakage was normalized with MTT OD values, as previously reported [25].

### 2.6. Flow Cytometry Analysis of ROS Release

HaCaT cells were seeded in 6-well plates at a density of 180,000 cells per well and treated with SS (1:40, 1:60, 1:80) and GA (4.1, 2.73, and 2.05 mM) for 6 and 18 h. Following treatment, cells were incubated with 5 μM of 5-(and-6)-chloromethyl-2′, 7′-dichlorodihydrofluorescein diacetate and acetyl ester (CM-H_2_DCFDA; Cat. C6827, Molecular Probes, Invitrogen, Life Sciences Division, Milan, Italy) for 1 h at 37 °C, as previously described [26]. CM-H_2_DCFDA is a cell-permeable fluorescent probe that, upon oxidation by intracellular ROS, emits green fluorescence. Fluorescence intensity was measured using a CytoFLEX flow cytometer (Beckman Coulter, Miami, FL, USA) equipped with an FL1 detector operating in logarithmic mode. The median fluorescence intensity (MFI) was calculated using CytExpert software (v2.3) to quantify ROS production. Dead cells were excluded from analysis by staining with Propidium Iodide (PI; 5 μg/mL, Cat. P4864, Sigma-Aldrich, St. Louis, MO, USA). A minimum of 10,000 events per sample was acquired for analysis.

### 2.7. Western Blot Analysis

HaCaT protein expression was measured after 14 and 24 or 48 h of treatment with SS (1:40, 1:60, 1:80) and GA (4.1 mM). Detached cells were centrifuged at 1200 rpm for 10 min at 4 °C to obtain the cell pellet. RIPA buffer with freshly added protease inhibitors (sodium orthovanadate at 1 mM, leupeptin at 50 µg/mL, aprotinin at 10 µg/mL and PMSF (Phenylmethyl sulfonyl fluoride) at 100 µg/mL, Merck Life Science, Milan, Italy), was used to suspend the pellet. The suspended pellet was then centrifuged at 15,000× *g* for 15 min at 4 °C and supernatant was collected for protein quantification using the Bicinchoninic acid assay (QuantiPro™ BCA Assay kit for 0.5–30 µg/mL protein, Merck Life Science, Milan, Italy) following the manufacturer’s instructions. A total of 25 µg of protein was separated on an 8% and 12% (sodium dodecyl sulfate (SDS))-polyacrylamide gel by electrophoresis and transferred to a nitrocellulose membrane. The membranes were saturated with 5% non-fat milk or 3% Bovine Serum Albumin (BSA). The membranes were incubated overnight at 4 °C under gentle shaking with specific primary antibodies, including anti-PI3K (rabbit, 1:1000), anti-p-Akt (rabbit, 1:1000), anti-Akt (rabbit, 1:1000), (Cell Signaling Technology, Danvers, MA, USA) anti-Nf-kβ (rabbit, 1:800) (Santa Cruz Biotechnology, Inc., Dallas, TX, USA), anti-COX2 (mouse, 1:1000) (Cayman Chemical, Ann Arbor, MI, USA), and anti-Tubulin (mouse, 1:1000) (Merck Life Science, Milan, Italy). On the next day, membranes were washed thrice with PBS tween−20 and membranes were incubated with specific IgG horseradish peroxidase (HRP)-conjugated secondary antibodies (Calbiochem, Darmstadt, Germany). Immunoreactive bands were detected using LiteAblot Extend Chemiluminescent Substrate (EuroClone S.p.a., Milan, Italy). The density of each band was identified using the ChemiDoc™ XRS system, and the QuantityOne 1-D analysis software, v 4.6.6 (BIORAD, Richmond, CA, USA), was applied to conduct densitometric analysis. Densitometric values of each band were normalized with OD values of internal tubulin.

### 2.8. RNA Extraction

HaCaT cells were collected from 6-well plates after 6 and 24 h of treatment and centrifuged at 1200 rpm to obtain cell pellets. The cells were lysed by 1% 2-mercaptoethanol lysis buffer followed by the addition of same volume of 70% ethanol (*v*/*v*) prepared in RNase-free water. Samples were transferred to spin cartridges, provided by the PureLink^®^ RNA Mini Kit (Life Technologies, Carlsbad, CA, USA); to avoid DNA contamination, before RNA extraction, samples were incubated for 15 min with 80 µL of DNase mixture (On-column PureLink^®^ Dnase Treatment, Life Technologies, Carlsbad, CA, USA). RNA was then eluted in 30 µL of RNase free-water and concentrations were measured (ng/µL) using the Qubit^®^ RNA BR Assay kit using a Qubit^®^ 4.0 Fluorometer reader (ThermoFisher Scientific Waltham, MA, USA).

### 2.9. Reverse Transcription and Real-Time Polymerase Chain Reaction (RT-PCR)

A high-capacity cDNA reverse transcription kit (Life Technologies, Carlsbad, CA, USA) was used for reverse transcription. For each sample, 360 ng of RNA was reverse-transcribed by mixing them in a 2X-RT master mix and incubating them in a thermal cycler (25 °C for 10 min, 37 °C for 120 min, and 85 °C for 5 min) to obtain cDNA. For assessing gene expression by quantitative PCR, the PowerUp™ SYBR™ Green 2X Master Mix (Thermo Fisher Scientific, Waltham, MA, USA) was used. The reaction volume (10 ng of cDNA, 1 µM of each forward and reverse primer, 10 µL of SYBR Green 2X Master Mix, and RNase free water to make up final volume of 20 µL) was transferred to a MicroAmp^®^ Optical 96-well plate (Life Technologies, Carlsbad, CA, USA) for amplification. The sequence of primers is listed in Table 1.

The reaction plate was then loaded in QuantStudio 3.0 (Thermo Fisher Scientific, Waltham, MA, USA) and the amplification method was set according to the instructions of the supplier: 50 °C and 95 °C each for 2 min, followed by the 40 cycles of amplification at 95 °C for 15 s, and 60 °C for 60 s. QuantStudio™ Design and Analysis Software v1.5.1 (Thermo Fisher Scientific, Waltham, MA, USA) was employed for gene expression data analysis. Melt curve analysis confirmed the authenticity of the PCR products. Values of gene expression were normalized using glyceraldehyde 3-phosphate dehydrogenase (GAPDH) levels. For each time point, the fold changes in target genes were calculated relative to GAPDH expression. Relative mRNA abundance was quantified using the comparative 2^−∆∆Ct^ method.

### 2.10. IL-6 Secretion ELISA Assay

After 48 h of treatment with SS or GA, the amount of IL-6 released in the supernatants for each condition was detected by using an IL-6 ELISA kit (Enzo Life Sciences, Farmingdale, NY, USA) following the manufacturer’s instructions. The absorbance was spectrophotometrically measured at 450 nm (Varioskan™ LUX Multimode Microplate Reader, Thermo Scientific, Waltham, MA, USA). The concentration of IL-6, expressed as pg/mL, was calculated thanks to an interpolation on the standard curve and then normalized on the MTT data.

### 2.11. Wound Healing Assay

The wound healing assay was performed on EA.hy926 endothelial cells and HaCaT keratinocytes by the normal scratch method. A scratch was made in the center of each well using a 200 µL micropipette tip. Dead and scratched cells were washed with normal media (DMEM) and adherent cells were treated with SS (concentrations: 1:40, 1:60, 1:80) and GA (concentration: 4.1 mM) in DMEM while the control was obtained without SS. To specifically assess cell migration independently of proliferation, the scratch assay was performed in serum-free medium (FBS-free). Photos were taken using an inverted light microscope (Leica DMi1, Wetzlar, Germany) equipped with a camera, using Leica LAS EZ software (LAS EZ 3.4 DVD 272), at different time intervals (0 h and 18 h of treatment for endothelial cells and 0 h, 24 h, and 48 h for keratinocytes). The scratch distance and area were measured using Fiji imageJ 1.54f software. The percentage of cut reduction after treatment was then normalized to the cut at T0.

### 2.12. Statistical Analysis

Statistical analysis was performed using GraphPad Prism v.8.0 software (San Diego, CA, USA) using one-way and two-way ANOVA (Analysis of variance) followed by Dunnett’s post hoc test. The data were presented as the mean ± SD (standard deviation), and *p* < 0.05 was considered statistically significant.

## 3. Results

### 3.1. Assessment of HaCat Viability After SS and GA Treatment

**Figure 1:**
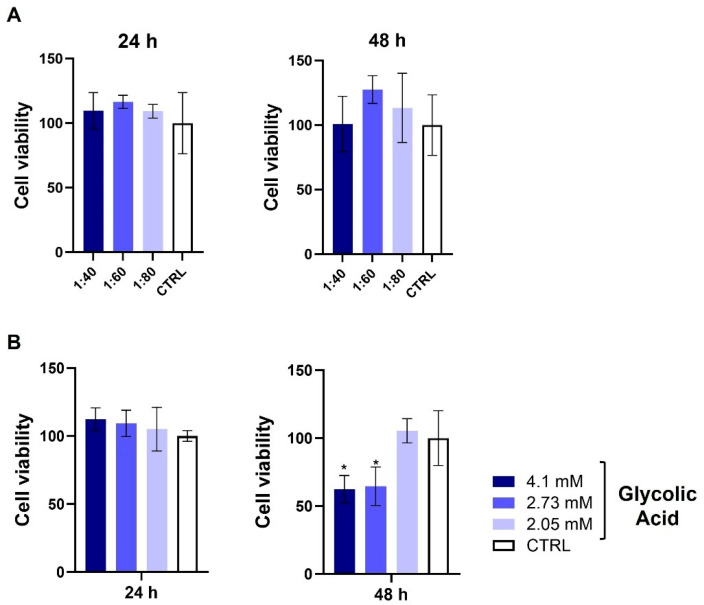
Cell viability test (MTT) was performed on HaCaT cells exposed to SS (1:40, 1:60, and 1:80 dilutions) (**A**) and GA (4.1, 2.73, and 2.05 mM) (**B**) for 24 and 48 h. Data are expressed as percentage of viable cells relative to control. Bars represent mean ± SD of three independent experiments. * *p* < 0.05 vs. CTRL.

HaCaT viability was assessed using the MTT assay after 24 and 48 h of treatment with different concentrations of SS (1:40, 1:60, and 1:80) and of GA (4.1, 2.73, and 2.05 mM, the same concentrations as those present in SS) and compared to that of the untreated control. At both time points, SS-treated cells showed no significant differences in viability compared to the control. In contrast, GA-treated cells exhibited no significant change after 24 h; however, after 48 h, treatment with GA at 4.1 and 2.73 mM significantly reduced cell viability compared to that of the control, as illustrated in Figure 1B.

### 3.2. Evaluation of GA’s and SS’s Effects on HaCaT Cytotoxicity

**Figure 2:**
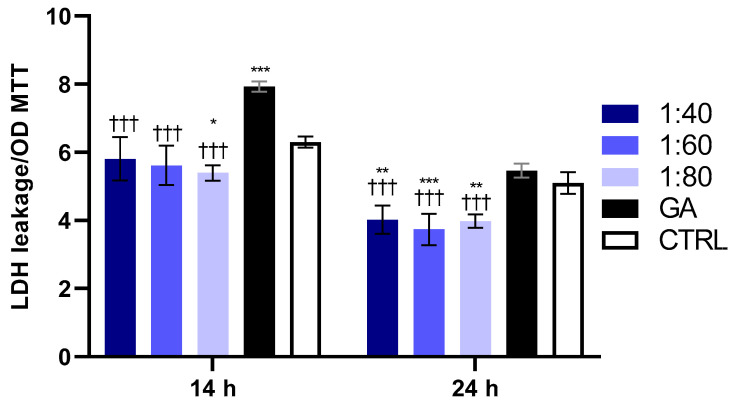
LDH release in HaCaT cells exposed to SS at 1:40, 1:60, and 1:80 dilutions and to GA (4.1 mM) for 14 and 24 h. LDH leakage values were normalized on MTT OD values. Data are presented as mean ± SD of three independent experiments; 14 h: * *p* < 0.05 vs. CTRL; *** *p* < 0.01 vs. CTRL; ††† *p* < 0.001 vs. GA; 24 h: ** *p* < 0.01 vs. CTRL; *** *p* < 0.001 vs. CTRL; ††† *p* < 0.001 vs. GA.

At both experimental time points (14 and 24 h), SS significantly reduced LDH release at all tested dilutions (1:40, 1:60, and 1:80) compared to both GA and the control (Figure 2). In contrast, GA provoked a significant increase in LDH release after 14 h compared to the control.

### 3.3. Measurement of ROS Level After SS and GA Treatment

**Figure 3:**
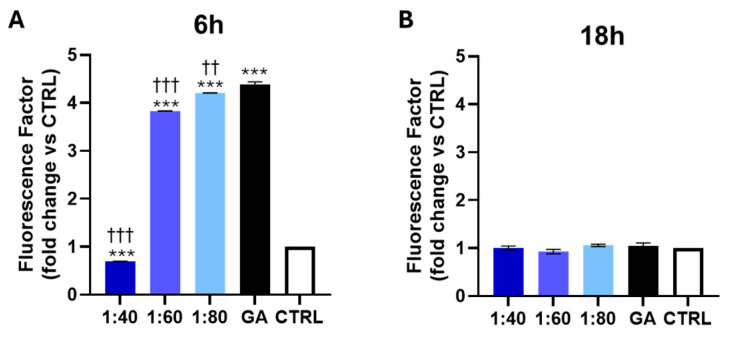
ROS levels measured by flow cytometry after 6 (**A**) and 18 h (**B**) of treatment with SS at 1:40, 1:60, and 1:80 dilutions and with GA (4.1 mM). Histograms represent median values ± SD of three independent experiments in which the Y axis reports mean fluorescence intensity (MFI) generated by the oxidation of H2DCFDA (generation of intracellular ROS) and is reported as fold change vs. CTRL. *** *p* < 0.001 vs. CTRL; ††† *p* < 0.001 vs. GA; †† *p* = 0.01 vs. GA.

To evaluate the effect of SS and GA treatments on ROS production, a flow cytometry analysis was performed using H2DCFDA-labeled HaCaT cells. After 6 h of treatment, 1:60 and 1:80 SS dilutions, as well as GA, significantly increased ROS levels compared to the control, while 1:40 dilution led to a reduction in ROS production. Interestingly, all SS-treated cells exhibited lower ROS levels compared to GA-treated cells (Figure 3A). After 18 h, a plateau in ROS levels was observed, with no significant changes detected in ROS levels between the SS- or GA-treated groups and the control, nor among cells treated with different SS dilutions compared to GA-treated cells (Figure 3B).

### 3.4. Effect of SS and GA Treatment on PI3K/Akt/NF-κB Protein Levels

**Figure 4:**
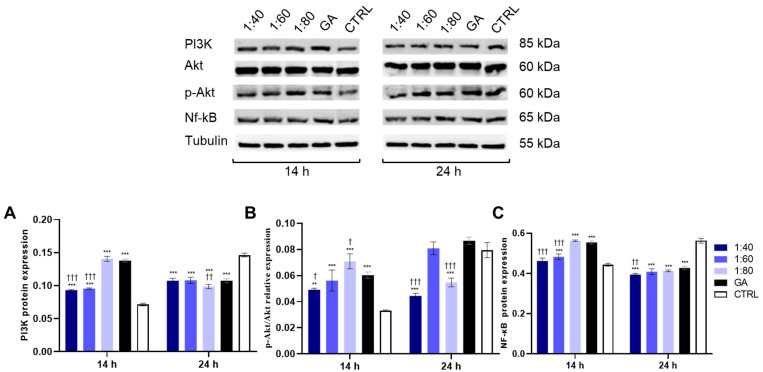
Western blotting analysis of PI3K (**A**), Phospho-Akt (p-Akt) and Akt (**B**), and NF-kB (**C**) expression levels in HaCaT cells treated with SS at 1:40, 1:60, and 1:80 dilutions and with GA (4.1 mM) for 14 and 24 h. The graph represents a densitometric analysis of protein expression levels normalized on tubulin expression levels. Data are presented as the mean ± SD of three independent experiments. A: 14 h: *** *p* < 0.001 vs. CTRL; ††† *p* < 0.001 vs. GA; 24 h: *** *p* < 0.001 vs. CTRL; †† *p* < 0.01 vs. GA. B: 14 h: ** *p* < 0.01 vs. CTRL; *** *p* < 0.001 vs. CTRL; † *p* < 0.05 vs. GA; 24 h: *** *p* < 0.001 vs. CTRL; ††† *p* < 0.001 vs. GA. C: 14 h: *** *p* < 0.001 vs. CTRL; ††† *p* < 0.001 vs. GA; 24 h: *** *p* < 0.001 vs. CTRL; †† *p* < 0.01 vs. GA, For the original data of Western blot in Figure 4, please refer to Supplementary Materials Figures S1–S5 and Tables S1–S3.

The PI3K/Akt/NF-κB pathway is a key cascade activated in response to ROS generation during mild inflammatory states. Therefore, an evaluation of their protein expression levels was performed after 14 and 24 h of treatment with SS and GA. After 14 h, PI3K expression level significantly increases in all SS-treated groups (1:40, 1:60, 1:80) and in a GA-treated group (4.1 mM), compared to the control. However, a significant reduction was observed in 1:40 and 1:60 SS-treated samples compared to that in GA-treated cells. After 24 h, PI3K expression levels were significantly decreased in all SS- and GA-treated samples compared to those in the control. Notably, the 1:80 SS-treated sample showed a significant reduction compared to GA-treated cells, as shown in Figure 4A. Regarding the p-Akt/Akt expression ratio, 14 h was sufficient to significantly increase the ratio in all SS- and GA-treated samples compared to that in the control. Specifically, 1:40 SS treatment resulted in a significant reduction in the p-Akt/Akt ratio compared to GA, while 1:80 SS treatment showed a significant increase compared to GA. After 24 h, both the 1:40 and 1:80 SS-treated samples disclosed a significant reduction compared to both the control and GA-treated cells (Figure 4B). For NF-κB, protein expression significantly increased after 14 h of treatment with 1:60 and 1:80 SS and GA compared to that in the control, while a significant reduction in 1:40 and 1:60 SS-treated samples was detected with respect to GA-treated cells. After 24 h, a significant reduction in NF-κB expression was recorded in all SS- and GA-treated samples, with respect to the control, with the 1:40 SS-treated group showing a further significant decrease relative to the GA-treated group (Figure 4C).

### 3.5. Assessment of Inflammatory Response

**Figure 5:**
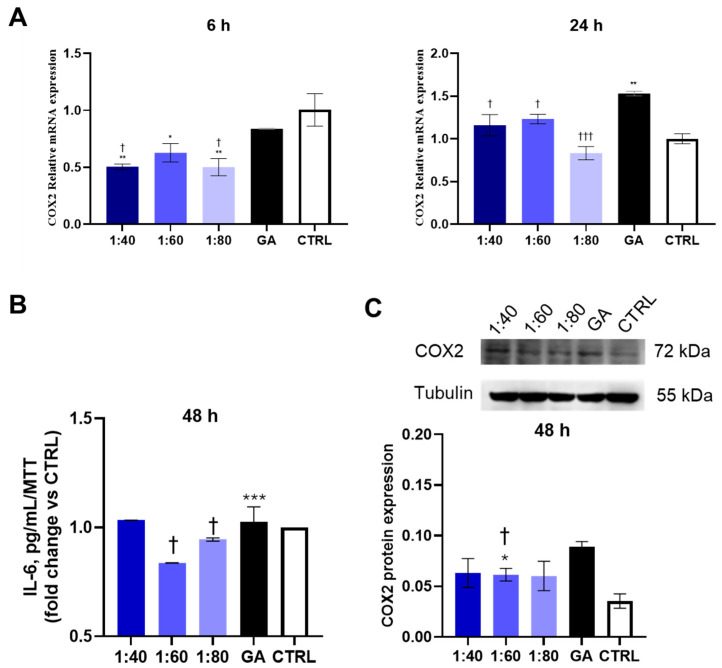
(**A**) COX2 gene expression in HaCaT cells treated with SS at 1:40, 1:60, and 1:80 dilutions and with GA (4.1 mM) for 6 and 24 h, analyzed by RT-PCR. COX2 gene expression was normalized with respect to GAPDH. Data are presented as mean ± SD; 6 h: ** *p* < 0.01 vs. CTRL; * *p* < 0.05 vs. CTRL; † *p* < 0.05 vs. GA; 24 h: ** *p* < 0.01 vs. CTRL; † *p* < 0.05 vs. GA; ††† *p* < 0.001 vs. GA. (**B**) COX2 protein expression levels in HaCaT cells treated with SS at 1:40, 1:60, and 1:80 dilutions and with GA (4.1 mM) for 48 h. The graph represents a densitometric analysis of protein expression levels normalized on tubulin. Data are presented as mean ± SD of three independent experiments. *** *p* < 0.001 vs. CTRL; † *p* < 0.05 vs. GA. (**C**) ELISA assay for IL-6 secretion after 48 h of SS and GA treatment. IL-6 levels are reported as pg/mL normalized on MTT and presented as fold change vs. CTRL. * *p* < 0.05 vs. CTRL; † *p* < 0.05 vs. GA. The original data of Western blot in Figure 5C, please refer to Supplementary Materials Figure S6 and Table S4.

In order to evaluate the inflammatory response of HaCaT cells after treatment with SS and GA, COX2 gene and protein expression was assessed. COX2 gene expression was analyzed after 6 and 24 h of treatment. After 6 h, all SS-treated samples showed a statistically significant decrease in COX2 gene expression compared to the control; moreover, 1:40 and 1:80 SS-treated samples showed a significant reduction in COX2 gene expression, also compared to GA-treated cells. After 24 h, GA-treated sample revealed a significant increase in COX2 gene expression with respect to the control while all the SS-treated samples (1:40, 1:60, 1:80) disclosed a significant reduction compared to GA-treated cells (Figure 5A). COX2 protein expression was evaluated after 48 h of treatment. GA induced a statistically significant increase in COX2 protein expression compared to the control, while no statistically significant changes were recorded comparing all SS dilutions with the control. In addition, 1:60 and 1:80 SS showed a significant reduction in protein expression with respect to GA (Figure 5B).

In order to consolidate these findings, a measurement of the release of pro-inflammatory cytokine IL-6 within the supernatant, after 48 h of treatment with SS and GA, was carried out by means of ELISA. The 1:60 condition produced a reduction statically significant with respect to both the control and GA (Figure 5C).

### 3.6. Analysis of MMP Gene Expression in Response to SS and GA Treatment

**Figure 6:**
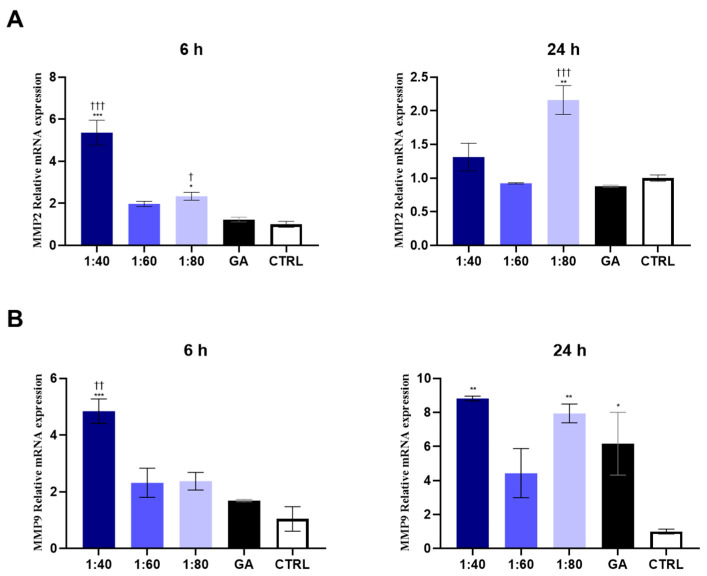
MMP-2 (**A**) and MMP-9 (**B**) gene expression in HaCaT cells treated with SS at 1:40, 1:60, and 1:80 dilutions and with GA (4.1 mM) for 6 and 24 h, analyzed by RT-PCR. Data are normalized on GAPDH and are expressed as mean ± SD of three independent experiments. (**A**) 6 h: *** *p* < 0.001 vs. CTRL; * *p* < 0.05 vs. CTRL; ††† *p* < 0.001 vs. GA; † *p* < 0.05 vs. GA; 24 h: ** *p* < 0.01 vs. CTRL; ††† *p* < 0.001 vs. GA. (**B**) 6 h: *** *p* < 0.001 vs. CTRL; †† *p* < 0.01 vs. GA; 24 h: ** *p* < 0.01 vs. CTRL; * *p* = 0.05 vs. CTRL.

In order to investigate the effect of SS and GA on ECM remodeling, an evaluation of MMPs was conducted in HaCaT cells after 6 and 24 h of treatment. In particular, MMP-2 and MMP-9 gene expression was measured. After 6 h, 1:40 and 1:80 SS-treated samples showed a statistically significant increase in MMP-2 gene expression, with respect to the control and GA-treated cells. After 24 h, only the 1:80 SS-treated sample maintained a significantly higher level of MMP-2 compared to the GA-treated cells and control (Figure 6A).

After 6 h, the 1:40 SS-treated sample disclosed an increased MMP-9 gene expression compared to the control and GA-treated cells, while after 24 h, the 1:40 and 1:80 SS- and GA-treated samples revealed a significant increase compared to the control (Figure 6B).

### 3.7. Wound Healing Assessment in HaCaT Cells After SS and GA Treatment

**Figure 7:**
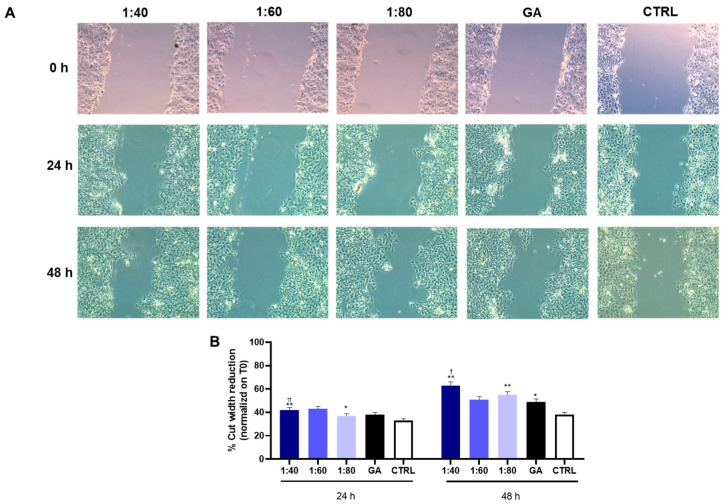
Wound healing assay in HaCaT cells after treatment with SS at different concentrations (1:40, 1:60, 1:80) and GA (4.1 mM). (**A**) Representative images are reported, acquired at 10X magnification. (**B**) Bar graph displays the percentage of cut width reduction after 24 h and 48 h of treatment, normalized on the cut measurement at T0. * *p* < 0.05 vs. CTRL; ** *p* < 0.01 vs. CTRL; † *p* < 0.05 vs. GA; †† *p* < 0.01 vs. GA.

To investigate the effects of SS and GA on keratinocyte migration—a key step in epidermal regeneration—a wound healing assay was performed using HaCaT cells. A scratch was introduced in each well to simulate a wound, and baseline images were captured at 0 h. Cells were then treated with SS (1:40, 1:60, 1:80) or GA (4.1 mM), and wound closure was assessed after 24 and 48h h by comparing the residual wound area to the baseline area. Results demonstrated that SS promoted HaCaT cell migration in a dilution-dependent manner. After 24 h and 48 h of treatment, both the 1:40 and 1:80 SS dilutions showed a significantly higher percentage of wound closure compared to the control. Although GA treatment also led to a significant increase in wound closure after 48 h, its effect remained lower than that observed with the 1:40 SS dilution at both time points. (Figure 7). These findings further support the regenerative potential of SS over isolated GA in promoting re-epithelialization.

### 3.8. Evaluation of Endothelial Cell Viability

**Figure 8:**
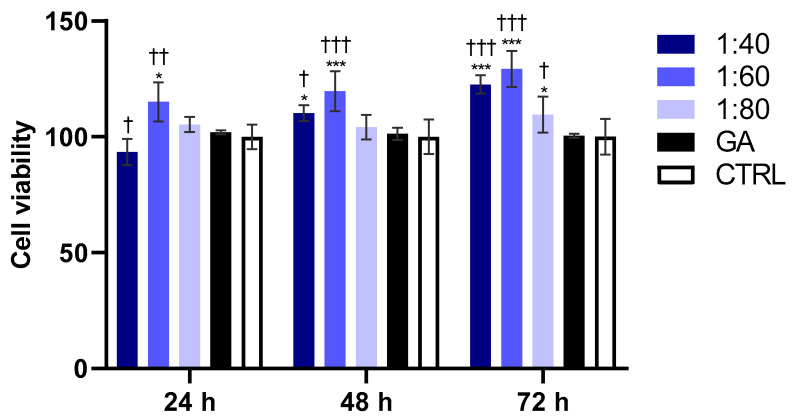
MTT assay performed on endothelial cell lineage (EA.hy926) after treatment with 1:40, 1:60, and 1:80 SS dilutions and with GA (4.1 mM) for 24, 48, and 72 h. Percentage of viable cells was normalized with control treated with DMEM and adjusted at 100%. Data are shown as mean ± SD of three independent experiments. For 24 h: * *p* < 0.01 vs. CTRL; † *p* < 0.05 vs. GA; †† *p* < 0.01 vs. GA; for 48 h: * *p* < 0.05 vs. CTRL; *** *p* < 0.001 vs. CTRL; † *p* < 0.05 vs. GA; ††† *p* < 0.001 vs. GA; for 72 h: * *p* < 0.05 vs. CTRL; *** *p* < 0.001 vs. CTRL; † *p* < 0.05 vs. GA; ††† *p* < 0.001 vs. GA.

The percentage of metabolically active EA.hy926 cells was assessed using the MTT assay after 24, 48, and 72 h of treatment with SS and GA. After 24 h, cells exposed to the 1:40 SS dilution recorded a significant viability reduction compared to GA-treated cells, while the 1:60 dilution showed a significant increase in cell viability compared to GA-treated cells and the control. At 48 h, 1:40 and 1:60 treated-cells showed a significant increase in cell viability compared to GA-treated cells and the control. Notably, after 72 h, all the SS dilutions, 1:40, 1:60, and 1:80, recorded a significant improvement in cell viability with respect to GA-treated cells and the control (Figure 8).

### 3.9. Wound Healing Assessment in Endothelial Cells After SS and GA Treatments

**Figure 9:**
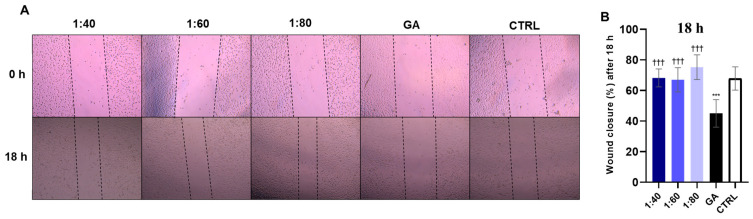
Wound healing assay in EA.hy926 cells after treatment with SS at different concentrations (1:40, 1:60, 1:80) and GA (4.1 mM). (**A**) Representative images are reported, acquired at 5× magnification. (**B**) Bar graph displays percentage of cut width reduction after 18 h of treatment, normalized on cut measurement at T0. *** *p* < 0.001 vs. CTRL; ††† *p* < 0.001 vs. GA.

The wound healing assay was performed on endothelial EA.hy926 cells in order to investigate the migratory capability of the cells after SS and GA exposure. A scratch was made in each well of cultured cells and photos of the cut were taken before starting the treatment (0 h). After 18 h of treatment with SS and GA, photos were retaken to analyze cell migration by measuring the dimensions of the cut. Data was analyzed to estimate the percentage of wound closure after 18 h; results showed that all the SS dilutions (1:40, 1:60, 1:80) significantly improved the cell migration and filled the gap compared to GA, while a statistically significant reduction in wound closure is seen in GA-treated cells compared to the control (Figure 9).

## 4. Discussion

SS has extensively been used for treating inflammation, burns, and other skin disorders since ancient times. Its traditional therapeutic applications have triggered researchers to make contributions to advancing and exploring the beneficial effects of this unique secretion. Researchers have been working to delve into the use of SS in cosmetics and sunscreen products [27], as well as its antibacterial, anti-inflammatory and wound-healing activities across different dosage forms [28,29,30,31]. In contemporary research, particular attention has been given to the development of SS-loaded patches and nanoparticles for targeted drug delivery, aiming to treat a range of diseases and biological disorders [32,33,34].

Based on this knowledge, the present work aims at investigating three points: (I) an in-depth analysis of SS’s role on the outermost skin layer, represented by keratinocytes; (II) a comparative evaluation of GA, one of the main components of SS, when administered alone versus in its natural SS matrix, focusing on modulation of the pro-inflammatory response and ECM remodeling; (III) a preliminary evaluation of SS’s effect on EC viability and migration as a foundation for future in-depth investigation on deeper skin layers.

Firstly, an evaluation of SS’s tolerance and cytotoxicity on keratinocytes, the primary skin cell population exposed to topical applications, was required. This evaluation was performed by administering SS and GA alone. The latter is widely recognized as an abundant component of SS, mainly responsible for peeling and gentle exfoliating effects, promoting cell turnover, when applied at low concentrations and for a short period of time [35], by upregulating the synthesis of glycosaminoglycans and stimulating collagen production. The viability analysis revealed that SS appeared appreciably tolerated by keratinocytes at dilutions ranging from 1:40 to 1:80, while when administering GA alone at the same concentrations as those present within SS, a biological tolerance was admitted only after short exposure. In fact, differently from SS, GA exerted a keratinolytic effect when the exposure time was prolonged up to 48 h. This preliminary result led us to hypothesize that the complex composition of SS is able to mitigate the marked keratinolytic effect of GA, even when GA content is higher (dilution 1:40), probably thanks to many biologically active compounds. This hypothesis is further and strongly supported by cytotoxicity evaluation aiming at measuring the prompt release of LDH enzyme when cells face toxic stimuli. Our results, indeed, underline completely different behaviors in keratinocytes in the presence of SS and GA: in fact, it could be admitted that the beneficial composition of SS allows this product to be positively tolerated by keratinocytes, considerably reducing LDH release. Diametrically opposite is the biological response in the presence of GA: a considerable peak in enzyme release leads us to assume that, when administered alone, GA could trigger cytotoxic responses, which are totally reversed when GA is included in SS composition. This hypothesis aligns with earlier findings by Van Scott and colleagues who reported that, when applied for more than 10 min, GA possesses an intrinsic cytotoxicity. This is attributed to a possible deeper penetration into the skin and a conversion into free acid, thus showing disadvantages like apoptosis, pruritus, swelling, and erythema [36]. These results highlight a key advantage of SS: it retains the beneficial exfoliating properties of GA while minimizing cytotoxic effects, thereby avoiding excessive stress on epidermidis cells.

Keratinocytes undergo continuous regeneration and exfoliation. This process is essential for maintaining skin hydration and a youthful appearance, as it involves the shedding of dead cells and their replacement with newly formed cells [37]. If the balance between the regeneration of new cells and the elimination of dead cells is disturbed, it leads to inflammatory reactions causing, in some cases, skin disorders such as acne and melasma [38]. Thus, a physical peeling of the dead skin cells, alongside anti-inflammatory and antibacterial treatment, is necessary [39]. SS is a unique blend of multifunctional macromolecules that not only gently exfoliates dead epidermidis layers but also possesses anti-inflammatory properties, as widely demonstrated [30,34]. However, inflammation is also considered a physiological process with beneficial effects when it occurs for brief periods, aiming at triggering positive events such as activation of the immune system to fight off foreign invaders, remove debris, and heal injuries [40]. For instance, formulations containing GA are known to induce an initial temporary inflammatory process able to stimulate exfoliation and regeneration of the outermost skin layer [41]. Our results support the hypothesis that, when GA is applied alone, a pro-inflammatory and marked event occurs, and it seems to require a prolonged time to be resolved respect to the administration of GA in the SS mixture. This hypothesis emerged by analyzing the involvement of the PI3K/Akt/NF-κB pathway. Indeed, emerging evidence suggests that PI3K/Akt pathway is involved in skin development and homeostasis [42] through the activation of several intracellular molecules, such as NF-κB nuclear transcription factor. The latter, in fact, by activating further intracellular mediators, is a crucial regulator of numerous cellular events, such as proliferation, differentiation, migration, angiogenesis, and metabolism [43]. In particular, NF-κB is often associated with inflammatory responses: its translocation into the nucleus after specific stimuli promotes the expression of pro-inflammatory genes, such as interleukins, chemokines, TNF-α and others [44]. When a specific pro-inflammatory stimulus occurs, activated PI3K can phosphorylate Akt which, in turn, activates NF-κB inducing its translocation into the nucleus [45]. Our results suggest that when HaCaT cells were exposed to GA alone, the pro-inflammatory response could be activated: indeed, after 14 h of treatment, upregulation, disclosed by an evident similar trend for PI3K, Akt, and NF-κB proteins, was detected, thus suggesting a possible cascade activation. It can be evidenced that SS was also able to upregulate the aforementioned pathway, even if to a minor extent, thus inducing a brief inflammatory event. This assumption is further supported by the evidence that the experimental conditions in which PI3K, Akt, and the NF-κB pathway were mainly downregulated were those in which the highest quantities of SS were administered, thus reinforcing and supporting the hypothesis that the rich and complex composition of SS was able to attenuate the marked response obtained with GA alone. Liu et al. [43] described that a feedback mechanism interlinks the upregulation and the downregulation of NF-κB factor. In fact, the initial upregulation of NF-κB not only triggers pro-inflammatory pathways but also directly regulates cell-mediated immunity, survival, proliferation, cell cycle, and anti-apoptotic signals by inhibiting the c-Myc gene [46], inducing, in turn, cell protection mechanisms by inhibiting the necrosis followed by downregulation of NF-κB [44]. Our results are coherent with the findings of Liu and colleagues: an initial upregulation of NF-κB, presumably due to PI3K/Akt pathway recruitment, is then followed by a downregulation of the pathway, suggesting a possible resolution of the pro-inflammatory event and the beginning of a cell protection mechanism involving further molecular cascades. One of the key pro-inflammatory genes, whose expression is regulated by NF-κB, is COX-2 [47]. It has been already reported that COX-2 can regulate chemokine production by reducing the recruitment of pro-inflammatory cells and promoting the local accumulation of regulatory T cells, thus stimulating cell protection mechanism through the activation of cell-mediated innate immunity [48]. In addition, it was reported that skin wound healing requires a transient and tightly regulated induction of COX-2, which is essential for initiating the tissue repair process. However, prolonged or excessive inflammation can disrupt this balance, ultimately impairing healing and compromising skin integrity [49]. To assess the inflammatory effects of SS and GA, we investigated the expression of COX-2, a key pro-inflammatory enzyme, at both the gene and protein levels in HaCaT keratinocytes. Additionally, interleukin-6 (IL-6) release was evaluated as a further marker of inflammatory activation. Our results let us argue that the activated PI3K/Akt/NF-κB pathway, in the presence of GA alone, resulted in increased COX-2 gene expression after 24 h of treatment, confirming pro-inflammatory stimulation, which could be responsible, after a longer exposure, for cell viability reduction, as reported in the MTT after 48 h of treatment. Furthermore, COX-2 gene expression data support our initial hypothesis: the activation of the PI3K/Akt/NF-κB pathway after 14 h in the presence of SS was not reflected in increased COX-2 gene expression after 24 h, differently from what was detected in the presence of GA alone, thus underlining the role of SS in the attenuation of inflammation. Considering the results of PI3K/Akt/NF-κB protein expression and COX-2 gene expression, it can be concluded that both GA and SS were able to trigger a brief inflammation in keratinocytes, presumably recruiting the PI3K/Akt/NF-κB pathway. However, in the presence of GA, the inflammatory response was triggered immediately after the administration of GA, as demonstrated by COX-2 gene expression after 6 h, while SS required prolonged exposure to activate this response and, even when activated, it resulted in positive modulation. COX-2 protein expression measured at 48 h further confirmed that SS markedly modulated the inflammatory response in a time-dependent manner. GA induced a significant increase in COX-2 protein levels, highlighting its prolonged inflammatory potential. Conversely, none of the SS-treated groups showed a significant increase in COX-2 protein expression compared to the control. Notably, the 1:60 and 1:80 SS dilutions reduced COX-2 protein expression compared to GA, suggesting that SS not only prevents excessive inflammatory signaling but may also counteract GA-induced upregulation when administered as part of a complex mixture. To further validate these findings, we assessed IL-6 release after 48 h of treatment. Interestingly, when compared to GA, both the 1:60 and 1:80 dilutions exhibited lower IL-6 levels, supporting the conclusion that SS exerted a dampening effect on the inflammatory milieu. Altogether, these findings suggest that SS offers a controlled and modulated inflammatory response, in contrast to the more aggressive and sustained activation observed with GA. The ability of SS to attenuate key inflammatory mediators, such as COX-2 and IL-6, reinforces its potential as a safer and more biocompatible alternative for topical applications, particularly in formulations intended for sensitive or damaged skin. Moreover, the observed anti-inflammatory profile of SS supports its multifunctional role in wound healing, where a balanced inflammatory response is critical for successful tissue regeneration without scarring or chronic irritation [50].

In healthy skin, keratinocytes coordinate with fibroblasts to regulate skin homeostasis, particularly regulating ECM remodeling. In a paracrine way, keratinocytes stimulate fibroblasts to release ECM remodeling factors; at the same time, they are able to drive ECM reshaping themselves, according to Pfisterer et al. [51] and Russo et al. [52]. In the field of ECM remodeling, an essential role is played by gelatinases which, by degrading the organic components of the ECM, create a physical space for cell migration from the deeper layers of the skin to the surface ones [53], thus confirming the activation of MMPs as an essential step for cell migration [54]. MMP-2 (gelatinase A) and MMP-9 (gelatinase B), involved in collagen proteolysis and ECM removal, essential for wound reepithelization [55], are also able to accelerate cell migration, angiogenesis, cell cycle regulation, and connective tissue remodeling [56]. In fact, MMP-2 and -9 have important roles in accelerating cell migration and are expressed by keratinocytes at the leading edge of wounds to promote re-epithelialization, as discussed by Krishnaswamy et al. [57]. Additionally, gelatinases are the predominant enzymes found in higher amounts compared to other MMPs in chronic wound tissues and have broader substrate preferences than collagenases (MMP-1 and -13), thus degrading a wide gamut of ECM molecules. However, considering the role of other MMPs, such as the 1, 3, and 13 isoforms, in remodeling events, further studies are required to explore SS’s effect on the complete panel of MMPs [58,59]. This evidence is strongly supported by our experimental model in which the brief and initial inflammation was followed by subsequent MMP-2 and MMP-9 gene expression upregulation, thus pushing up ECM remodeling; this event appeared markedly more pronounced for SS than for GA alone, admitting that SS administration significantly sped up and sustained the ECM remodeling process.

Although ROS play a crucial role in signaling pathways and physiological processes, particularly in tissue repair, ROS function as a “double-edged sword”, beneficial when controlled but harmful when sustained [60]. The evaluation of intracellular ROS levels provides valuable insights into the oxidative status induced by topical agents such as SS and GA. The early ROS elevation in the 1:60 and 1:80 samples suggests that, at these dilutions, SS transiently stimulated oxidative signaling, which could act as a trigger for cellular processes such as proliferation and differentiation. Interestingly, the 1:40 SS dilution led to a reduction in ROS levels, indicating a potential antioxidant or ROS-scavenging effect at higher SS concentrations. These findings imply that components of SS may exert dual effects depending on the concentration, promoting redox signaling at moderate levels and exerting antioxidant activity at higher concentrations. GA treatment, in contrast, caused a marked and statistically significant increase in ROS production after 6 h, more pronounced than that with any SS dilution. This is consistent with GA’s known keratinolytic and exfoliative mechanisms, which are often accompanied by oxidative stress. The comparison between SS- and GA-treated cells supports the notion that the natural matrix of SS mitigates the oxidative stress associated with GA alone.

After 18 h of treatment, ROS levels in all groups (SS, GA, and control) converged; this plateau suggests the activation of cellular compensatory mechanisms and restoration of redox homeostasis over time. The absence of sustained ROS accumulation, especially in SS-treated cells, reinforces the idea that SS does not induce persistent oxidative stress, which is critical for avoiding chronic inflammation and cellular damage.

Overall, these findings highlight the redox-modulating properties of SS. Unlike GA, which triggers a stronger oxidative burst, SS induces a more controlled and transient ROS response that may support cellular activation without promoting prolonged oxidative damage. This behavior further emphasizes the potential of SS as a safer, well-tolerated alternative for skin regeneration therapies involving controlled oxidative stimuli.

Keratinocyte migration is a critical step in the re-epithelialization phase of wound healing and skin regeneration [61]. In the present study, a wound healing assay was employed to investigate the impact of SS and GA on keratinocyte migration. Our findings demonstrate that SS significantly enhanced HaCaT cell migration in a dose-dependent manner. Both the 1:40 and 1:80 dilutions of SS notably accelerated wound closure after 24 and 48 h of treatment. Although GA also significantly promoted wound closure at 48 h, its effect was considerably less pronounced than that of SS, particularly when compared to the 1:40 SS dilution, which maintained superior results at both 24 and 48 h. This discrepancy may be attributed to the limited functional scope of GA, which, while effective as a keratolytic agent, lacks the broader spectrum of bioactive molecules present in SS [62] that collectively support cell migration, proliferation, and ECM remodeling. The ability of SS to enhance keratinocyte migration more effectively than GA alone underscores its potential as a multifaceted regenerative agent in dermatological applications.

New blood vessel formation is a multistep and complex process, regulated by a tight balance between pro- and anti-angiogenic factors [63] and strictly connected to MMP activity, which is believed to promote degradation of the preexisting basement membrane and ECM components to control the release of angiogenic and growth factors, as well as to stimulate endogenous angiogenic inhibitors [64]. In addition, skin regeneration and reepithelization are processes requiring the activity of blood vessels, populating the dermis, similarly to the wound healing mechanism [65]. Based on this, an initial evaluation of SS’s effect on ECs was carried out in order to estimate SS’s capability to support an accurate skin regeneration process; thus, EC response, in terms of viability and migration, was assessed. Results showed that SS promoted EC viability throughout up to 72 h of treatment, compared to GA and the control. Moreover, the migratory capability of ECs, one of the main angiogenic factors in obtaining new blood network formation [66], was promoted when SS was administered. In fact, differently from GA, SS significantly improved cell migration and, interestingly, this effect appeared even more pronounced when SS was more diluted, achieving 75% wound closure compared to GA which achieved only 45%. This finding is in alignment with previous findings which already reported the valuable ability of SS in promoting angiogenesis during skin healing wounds [67].

## 5. Conclusions

This study underlines that SS, due to its unique blend of multiple beneficial macromolecules, appears well tolerated by skin cells. SS in comparison to GA does not impart cytotoxic burden to skin cells, while the latter does. Similarly, SS induces well-controlled and regulated brief inflammation, helpful for cellular recovery processes, while GA induces inflammation immediately after administration and requires a longer time to become restored, suggesting that the complex composition of SS is able not only to attenuate the strong effect of GA but also to counteract inflammation and to maintain normal skin homeostasis. In addition, SS promotes transient and controlled cellular activation rather than prolonged damage. This balanced response is evidenced by the downregulation of COX-2 and IL-6, alongside a temporary rise in intracellular ROS levels, which may play a role in initiating key regenerative processes such as keratinocyte migration. Moreover, this paper underlines SS’s ability to effectively promote ECM remodeling by significantly raising MMP expression. A promising effect is also exerted on ECs populating the deeper layer of the skin, making SS suitable for tailored preparations. Therefore, considering the complexity of the wound healing process and the involvement of the different tissues, our results add one more piece of evidence in understanding the role of SS in skin regeneration, allowing us to admit that SS is a potent agent in regulating cellular signaling pathways, and opens up future avenues for its potential use in wound healing. While our work provides mechanistic insights through in vitro assays, future studies employing animal models will be essential to validate these findings and assess wound closure dynamics, granulation tissue formation, and collagen deposition, thereby strengthening translational relevance. This positions our findings as a foundation for forthcoming translational research.

## Figures and Tables

**Table 1:** Sequence of primers used for qPCR gene expression.

Gene	Sequence (5′ to 3′)	Source
GAPDH-Fr	GGGTGTGAACCATGAGAAGTA	Primer blast
GAPDH-Rv	ACTGTGGTCATGAGTCCTTC	Primer blast
COX2-Fr	CCCTTCTGCCTGACACCTTT	Primer blast
COX2-Rv	TTCTGTACTGCGGGTGGAAC	Primer blast
MMP-2-Fr	GCTACGATGGAGGCGCTAAT	Primer blast
MMP-2-Rv	GGGCAGCCATAGAAGGTGTT	Primer blast
MMP-9-Fr	CGACGTCTTCCAGTACCGAG	Primer blast
MMP-9-Rv	GTTGGTCCCAGTGGGGATTT	Primer blast

## Data Availability

The raw data supporting the conclusions of this article will be made available by the authors on request.

## Supplementary Materials

The following supporting information can be downloaded at https://www.mdpi.com/article/10.3390/biom15091302/s1, Figure S1: Original nitrocellulose membrane; Figure S2: Original blots of tubulin and PI3K protein expression; Figure S3: Original blots of tubulin and p-AKT protein expression; Figure S4: Original blots of tubulin and AKT protein expression; Figure S5: Original blots of tubulin and Nf-kB protein expression Figure S6: Original blots of tubulin and COX-2 protein expression; Table S1: Original densitometric data of tubulin and PI3K Western blot, Table S2: Original densitometric data of tubulin and pAKT/AKT Western blot, Table S3: Original densitometric data of tubulin and Nf-kB Western blot, Table S4: Original densitometric data of tubulin and COX-2 Western blot.
