# Supplementary material for: Nature’s Synergy: Cellular and Molecular Evaluation of Snail Slime and Its Principal Component, Glycolic Acid, on Keratinocytes, with Preliminary Evidence from Endothelial Cells
Source: Biomolecules. 2025 Sep 10;15(9):1302. doi: 10.3390/biom15091302 (PMC12467389; doi:10.3390/biom15091302)
Supplement: Supplementary file 1 [file biomolecules-15-01302-s001.zip › WB bands.pptx]

## Slide 1
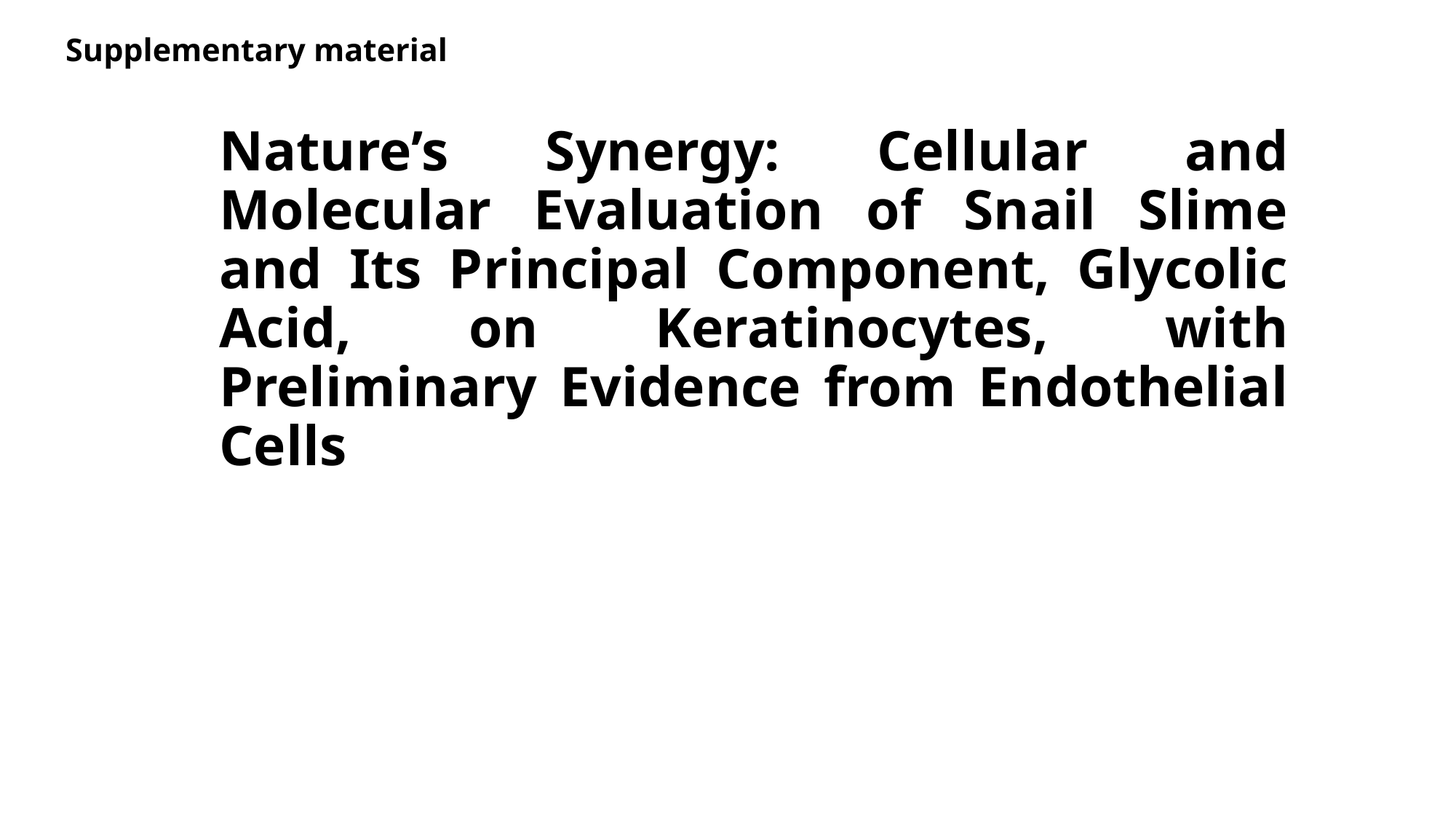

Supplementary material
# Nature’s Synergy: Cellular and Molecular Evaluation of Snail Slime and Its Principal Component, Glycolic Acid, on Keratinocytes, with Preliminary Evidence from Endothelial Cells

## Slide 2
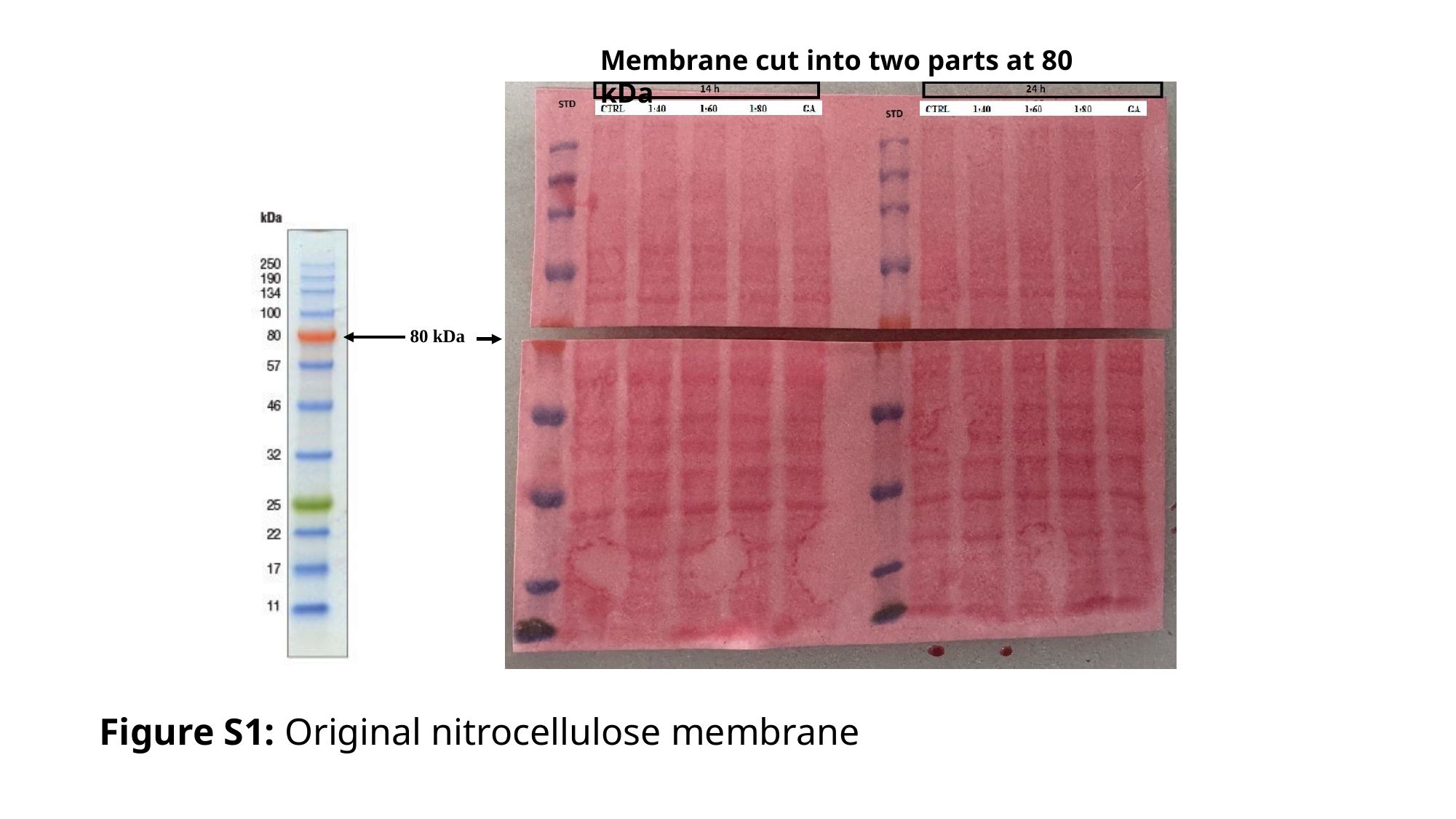

Membrane cut into two parts at 80 kDa
80 kDa
Figure S1: Original nitrocellulose membrane

## Slide 3
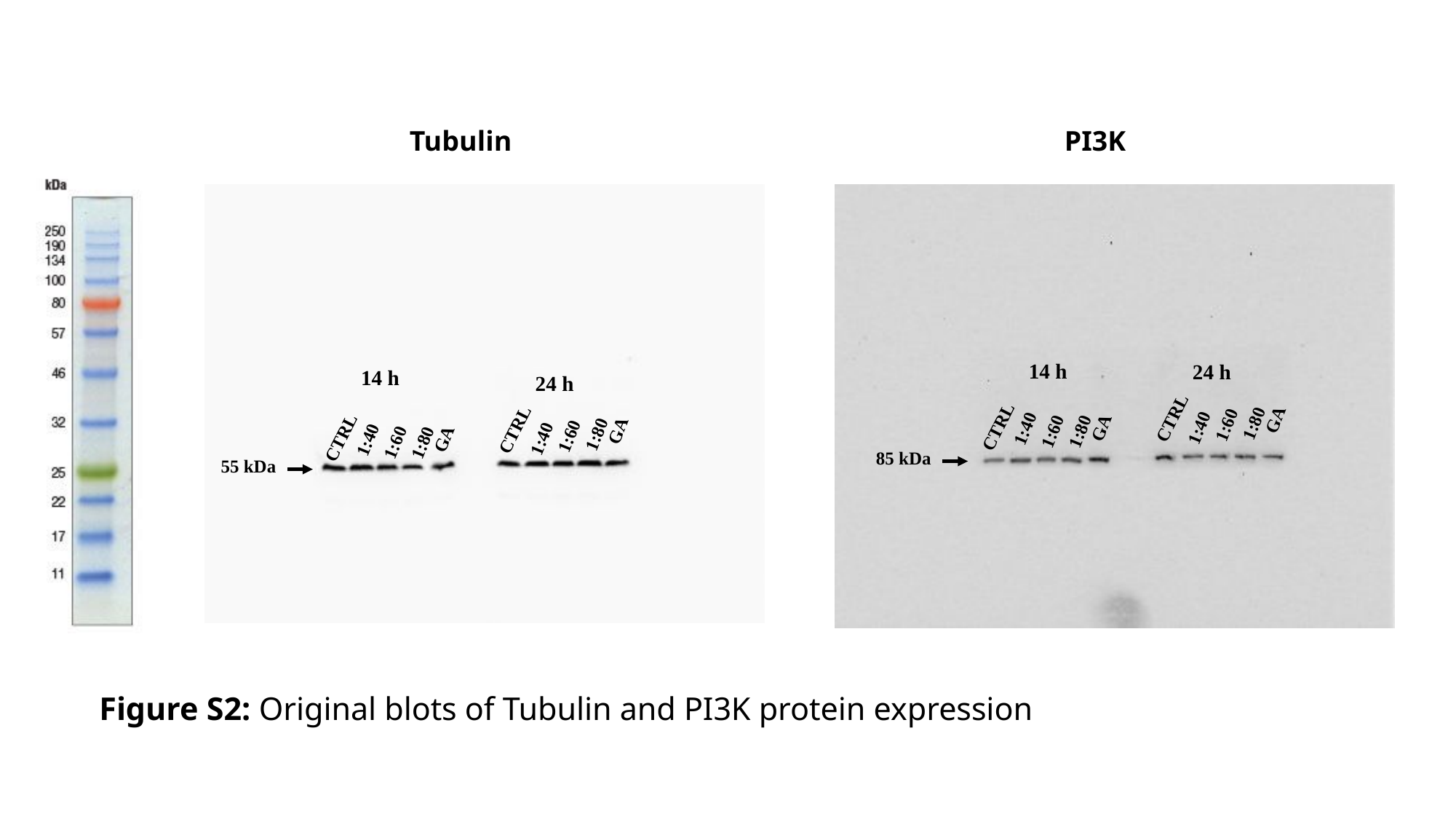

Tubulin						PI3K
14 h
24 h
GA
CTRL
1:80
1:60
GA
1:40
CTRL
1:40
1:80
1:60
14 h
24 h
GA
CTRL
1:80
1:60
GA
1:40
CTRL
1:40
1:80
1:60
85 kDa
55 kDa
Figure S2: Original blots of Tubulin and PI3K protein expression

## Slide 4
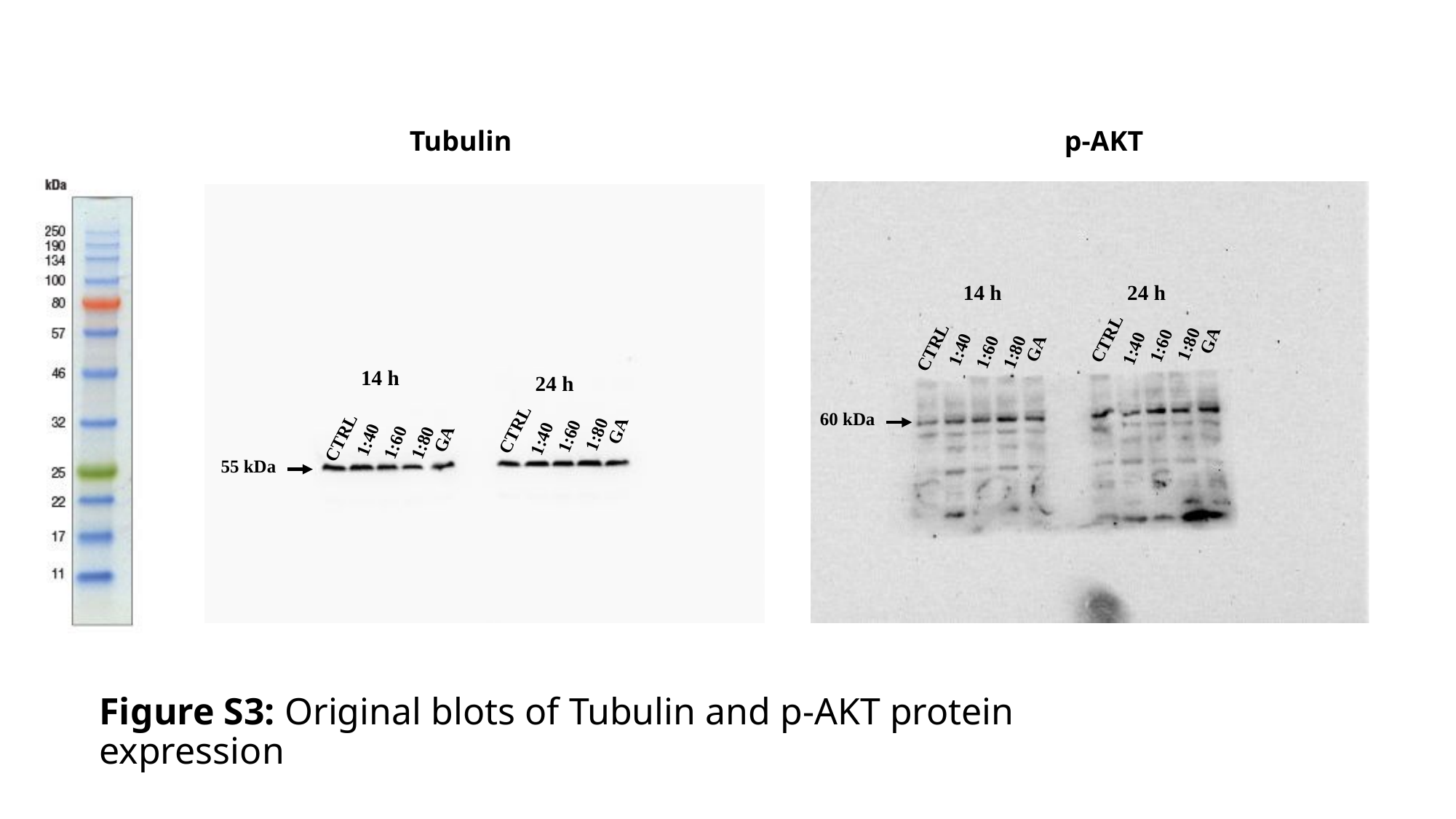

Tubulin						p-AKT
14 h
24 h
GA
CTRL
1:80
1:60
GA
1:40
CTRL
1:40
1:80
1:60
14 h
24 h
GA
CTRL
1:80
1:60
GA
1:40
CTRL
1:40
1:80
1:60
60 kDa
55 kDa
Figure S3: Original blots of Tubulin and p-AKT protein expression

## Slide 5
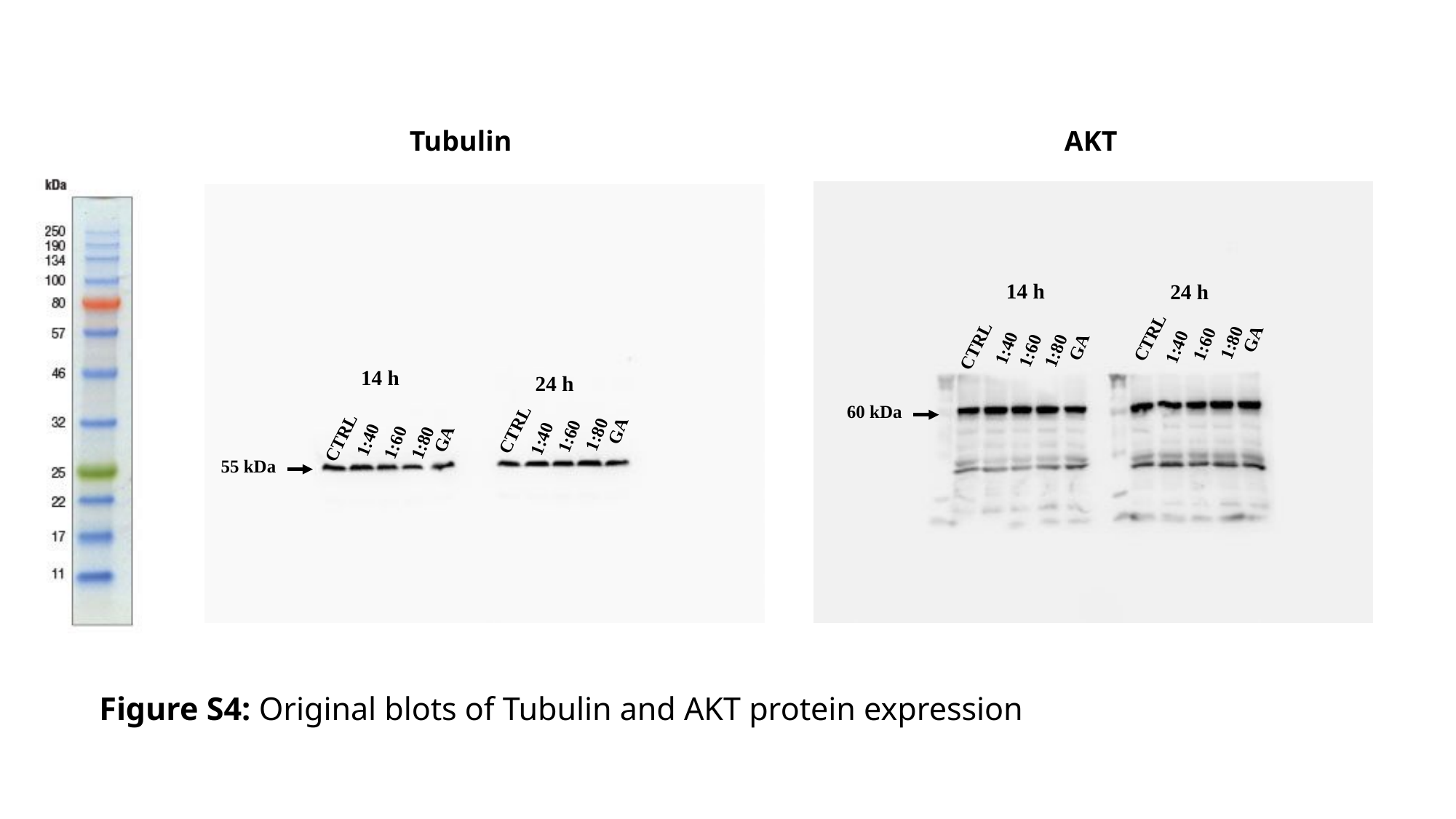

Tubulin						AKT
14 h
24 h
GA
CTRL
1:80
1:60
GA
1:40
CTRL
1:40
1:80
1:60
14 h
24 h
GA
CTRL
1:80
1:60
GA
1:40
CTRL
1:40
1:80
1:60
60 kDa
55 kDa
Figure S4: Original blots of Tubulin and AKT protein expression

## Slide 6
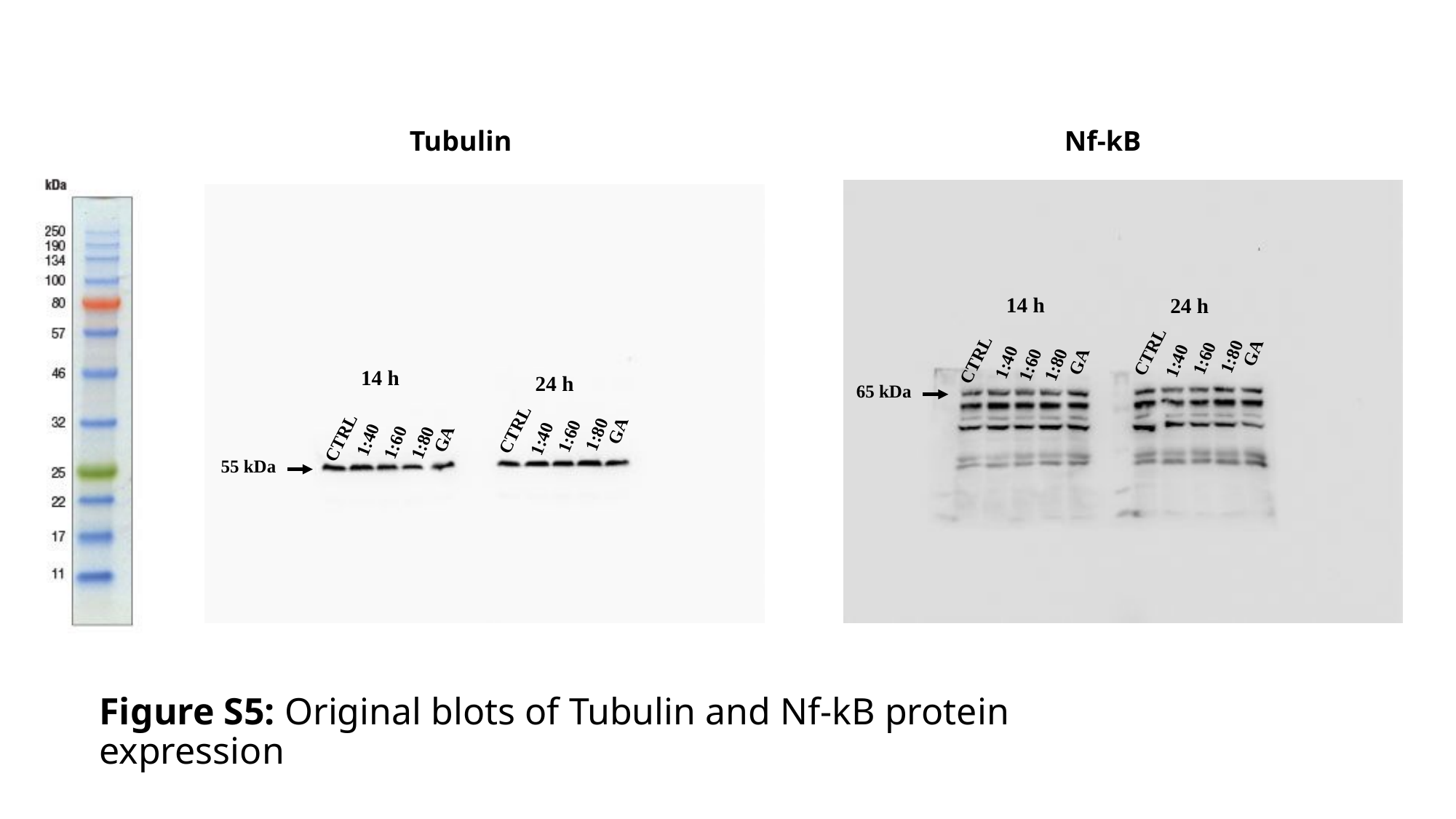

Tubulin						Nf-kB
14 h
24 h
GA
CTRL
1:80
1:60
GA
1:40
CTRL
1:40
1:80
1:60
14 h
24 h
GA
CTRL
1:80
1:60
GA
1:40
CTRL
1:40
1:80
1:60
65 kDa
55 kDa
Figure S5: Original blots of Tubulin and Nf-kB protein expression

## Slide 7
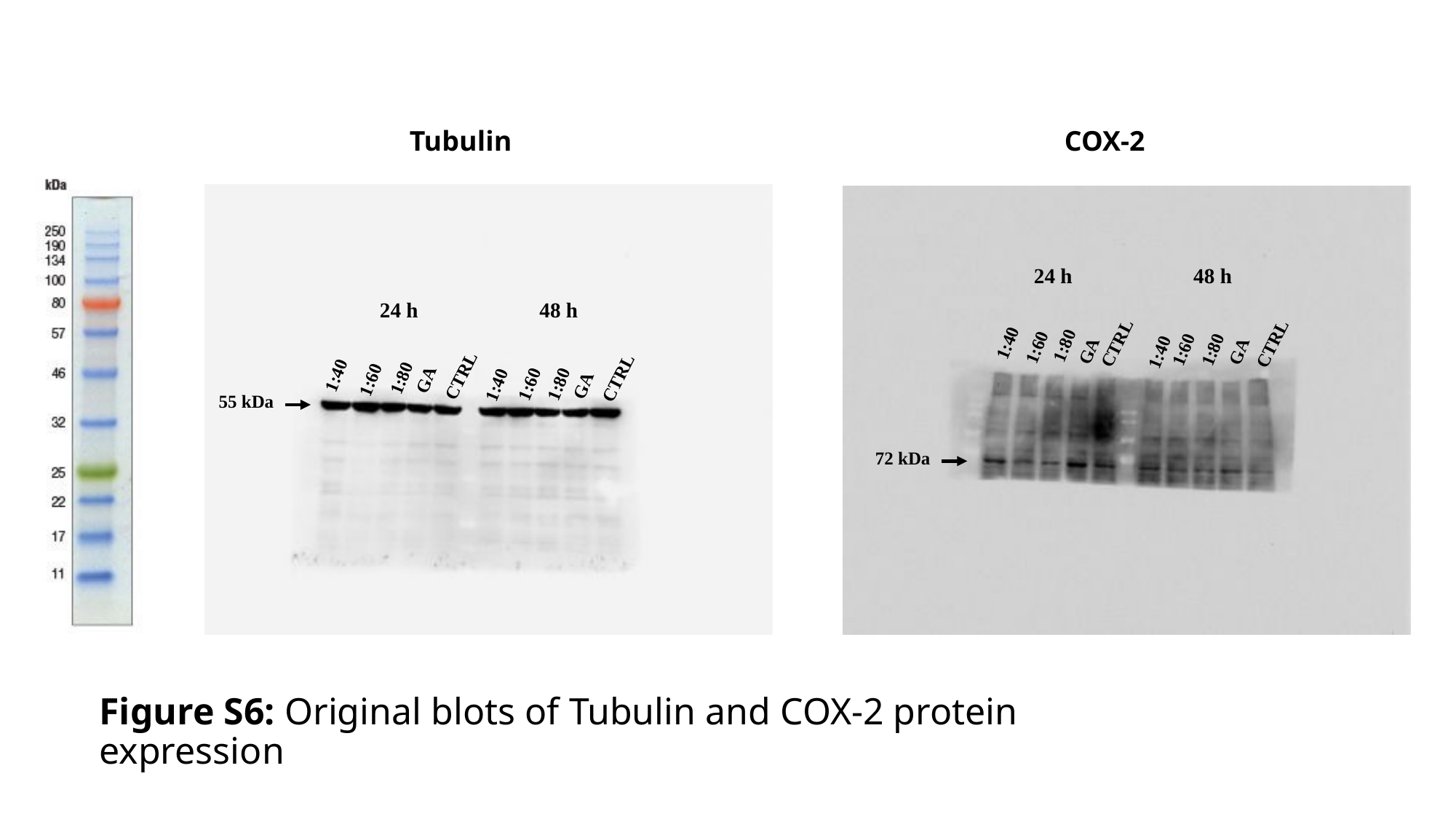

Tubulin						COX-2
24 h
48 h
1:40
CTRL
1:80
CTRL
1:60
GA
GA
1:80
1:60
1:40
24 h
48 h
1:40
GA
CTRL
1:80
1:60
CTRL
GA
1:80
1:60
1:40
55 kDa
72 kDa
Figure S6: Original blots of Tubulin and COX-2 protein expression
